# Cardiometabolic Risk in US Army Recruits and the Effects of Basic Combat Training

**DOI:** 10.1371/journal.pone.0031222

**Published:** 2012-02-23

**Authors:** Stefan M. Pasiakos, J. Philip Karl, Laura J. Lutz, Nancy E. Murphy, Lee M. Margolis, Jennifer C. Rood, Sonya J. Cable, Kelly W. Williams, Andrew J. Young, James P. McClung

**Affiliations:** 1 Military Nutrition Division, United States Army Research Institute of Environmental Medicine, Natick, Massachusetts, United States of America; 2 Pennington Biomedical Research Center, Louisiana State University System, Baton Rouge, Louisiana, United States of America; 3 Directorate of Basic Combat Training, Fort Jackson, South Carolina, United States of America; Pennington Biomedical Research Center, United States of America

## Abstract

**Background:**

Cardiometabolic disease risk in US military recruits and the effects of military training have not been determined. This study examined lifestyle factors and biomarkers associated with cardiometabolic risk in US Army recruits (209; 118 male, 91 female, 23±5 yr) before, during, and after basic combat training (BCT).

**Methodology/Principal Findings:**

Anthropometrics; fasting total (TC), high-density lipoprotein (HDL) and low-density lipoprotein (LDL) cholesterol; triglycerides (TG); glucose; and insulin were measured at baseline and every 3 wks during the 10 wk BCT course. At baseline, 14% of recruits were obese (BMI>30 kg/m^2^), 27% were cigarette smokers, 37% were sedentary, and 34% reported a family history of cardiometabolic disease. TC was above recommended levels in 8%, LDL in 39%, TG in 5%, and glucose in 8% of recruits, and HDL was below recommended levels in 33% of recruits at baseline. By week 9, TC decreased 8%, LDL 10%, TG 13%, glucose 6% and homeostasis model assessment of insulin resistance (HOMA-IR) 40% in men (*P*<0.05). In women, TC, LDL, glucose and HOMA-IR were decreased from baseline at weeks 3 and 6 (*P*<0.05), but were not different from baseline levels at week 9. During BCT, body weight declined in men but not women, while body fat percentage declined in both men and women (*P*<0.05).

**Conclusions/Significance:**

At the start of military service, the prevalence of cardiometabolic risk in US military recruits is comparable to that reported in similar, college-aged populations. Military training appears to be an effective strategy that may mitigate risk in young people through improvements in lipid profiles and glycemic control.

## Introduction

Cardiovascular and metabolic diseases remain leading causes of morbidity and mortality in the United States [1], [2]. Smoking, sedentary lifestyle, and poor dietary habits have been established as modifiable behaviors contributing to the development and progression of cardiometabolic disease in part by promoting dyslipidemia, elevated blood glucose, and overweight/obesity [3]–[7]. Studies investigating primary and secondary cardiometabolic disease prevention strategies have traditionally focused on modifying health risk behaviors in older adults [8]–[11]. However, cardiometabolic diseases are progressive in nature, and early indications of disease are evident in adolescents and young adults [12]–[17] with some reports suggesting that more than a third of young adults entering college already exhibit one or more risk factors for cardiometabolic disease [18], [19]. As exposure to cardiometabolic risk factors in childhood and adolescence is associated with disease development in adulthood [20]–[22] and health-risk behaviors are often established early in life, identifying strategies that deter the adoption and continuation of health risk behaviors in younger adults is essential for long-term primary prevention of cardiometabolic disease.

Military personnel are sometimes considered healthy, physically-fit adults that may be at low risk for developing cardiometabolic disease, as military service requires adherence to body composition, fitness [23], and medical standards [24]. However, evidence suggests that biomarkers and health-risk behaviors associated with cardiometabolic disease in military personnel may be similar to that observed in civilians [25], [26], with the rate of military personnel with a body mass index (BMI) exceeding 25 kg/m^2^ at 62% and the prevalence of dyslipidemia [26] and smoking [27] at approximately 30%. Further, as compared to historic data [25], military recruits are now less physically fit and heavier, with higher body fat, highlighting the necessity for effective primary prevention strategies.

Basic combat training (BCT) provides an opportunity for US Army Soldiers to adopt lifestyle behaviors that mitigate cardiometabolic disease risk. Lifestyle modifications include smoking and alcohol cessation, improved dietary habits through healthy food options at military dining facilities, and mandatory participation in standardized physical training. Whether the effects of military-specific physical training and behavioral modifications during BCT result in improvements in biomarkers of cardiometabolic disease risk in military recruits is undetermined. Therefore, this longitudinal study was conducted to characterize health-risk behaviors and cardiometabolic risk in US Army recruits at the start of BCT and to determine if lifestyle changes adopted during BCT influence biomarkers of disease risk. We hypothesized that cardiometabolic disease risk in US Army recruits would be consistent with that reported in college-aged adults and that completion of BCT would result in body fat loss, improved lipid profiles, and enhanced glycemic control.

## Materials and Methods

### Subjects

This study was approved by the Human Use Review Committee at the US Army Research Institute of Environmental Medicine and was conducted at Fort Jackson, SC. Human volunteers participated in the study after providing informed written voluntary consent. Investigators adhered to US Army Regulation 70-25 and US Army Medical Research and Material Command regulation 70-25 on the participation of volunteers in research.

US Army recruits who entered BCT during January 2010 volunteered to participate in this longitudinal trial. Anthropometrics, blood lipids, glucose, and insulin were assessed at baseline (wk 0) and again at wks 3, 6, and 9 of BCT. Demographics and health-risk behaviors (e.g., smoking and physical activity) associated with cardiometabolic risk were determined at wk 0 using health and family history questionnaires. A validated semi-quantitative food frequency questionnaire was administered at wk 0 and wk 9 to assess habitual dietary intake and changes in dietary intake during training (Block 2005 FFQ; Nutrition Quest, Berkeley, CA) [28], [29]. Non-plausible reporters, identified as men reporting an energy intake >5000 kcal/d or <800 kcal/d and women reporting energy intake >4500 kcal/d or <300 kcal/d, were excluded from the analysis.

### Basic Combat Training

US Army BCT is an integrated 10 wk physical and didactic military training program. Physical training includes a combination of aerobic-type exercise activities such as road marching with weighted loads, obstacle courses, distance running, and sprinting, as well as muscular strength- and endurance-type training (e.g., push-ups, sit-ups, and pull-ups). Military-related activities such as rappelling, weapons training, hand-to-hand combative training, and didactic classroom instruction are also required during the course. Although not directly assessed in the present study, estimates of physical activity during BCT have been previously reported [30]. Soldiers reside in supervised barracks and alcohol and smoking abstention is mandatory during BCT.

Dietary intake is self-selected during BCT and limited to food and beverages offered in military dining facilities. Soldiers are given a choice of one entre, starch, and vegetable from a selection of meal options that cycle daily. In addition, Soldiers are also able to choose from a variety of desert items, fruits, and beverages at each meal. Standard military rations are provided during field training exercises. All meals, including rations, are designed to meet Military Dietary Reference Intakes [31], and provided in accordance with the Army Food Program [32].

### Anthropometrics and Body Composition

Height was measured with a stadiometer (Creative Health Products, Plymouth, MI). Body mass was measured at each time point using a calibrated electronic scale (A&A Scales, Prospect Park, NJ). BMI was calculated from measured height and body mass. Skinfold thickness was measured at wks 0 and 9 at the chest, triceps and subscapular sites for men, and at the triceps, suprailiac and abdominal sites for females with Lange calipers (Beta Technology, Santa Cruz, CA) to estimate body composition [33]. Body fat percentage was derived from body density calculated using 3-site skinfold equations [34]–[36]. Body composition measurements were performed under similar experimental conditions (fasted, similar attire) by the same trained technician.

### Biological Analyses

Blood samples were collected after an overnight fast by antecubital venipuncture into tubes containing the appropriate anticoagulants (Vacutainer; Becton Dickson, Franklin Lakes, NJ). Serum was isolated, frozen, and shipped to the Pennington Biomedical Research Center (Baton Rouge, LA) for analysis of glucose, triglycerides (TG) and total (TC), low-density lipoprotein (LDL) and high-density lipoprotein (HDL) cholesterol using the Beckman Coulter DXC 600 Pro system (Beckman Coulter, Fullerton, CA). Insulin was assessed using an automated immunoassay instrument (Siemens Healthcare Diagnostics, Deerfield, IL); concentrations were used in the homeostasis model assessment of insulin resistance (HOMA-IR) [37].

### Statistical Analyses

Dichotomous variables were created to identify the prevalence of US Army recruits exhibiting health-risk behaviors and the percentage of recruits with BMI, body fat and serum lipid levels indicative of increased cardiometabolic risk at wk 0 and wk 9. Sex differences for dichotomous variables were assessed using Fisher's Exact test. Difference in prevalence between wk 0 and wk 9 was assessed using McNemar's test. Student's t-tests were used to identify sex differences in anthropometric variables, and glucose and serum lipid concentrations at wk 0. Two-factor (time and sex) mixed model repeated measures ANOVAs were used to examine changes in body mass, body fat percentage, glucose, HOMA-IR and serum lipid levels during BCT and to determine whether these changes differed by sex. Akaike's information criteria were used to determine appropriate covariance structures. Following observation of a significant sex-by-time interaction, post-hoc comparisons were completed using Bonferroni adjustments. Analyses were conducted using commercially available statistical software (SPSS 18.0; SPSS Inc., Chicago, IL). Descriptive statistics are presented as mean ± SD unless otherwise noted. Significance was set at *P*<0.05.

## Results

### Volunteer characteristics

Two hundred and nine US Army recruits [median age (yr) (interquartile range)], 118 males [21(19–25)], 91 females [21(19–24)] participated in this trial. Average BMI at week 0 was higher (*P*<0.05) for males (27.0±4.3 kg/m^2^) compared to females (25.0±2.9 kg/m^2^); however, percent body fat was lower (*P*<0.05) in males (14.2±4.6%) versus females (26.7±5.8%). Percent body fat for Soldiers with a BMI less than 30 kg/m^2^ was 13.0±3.9% in males and 26.3±5.5% in females. When BMI exceeded 30 kg/m^2^, percent body fat was 18.8±4.4% in males and 36.6±3.3% in females. In all cases, percent body fat was lower (*P*<0.05) in males. Volunteers (n) identified themselves as either white or Caucasian (72 males, 55 females), black or African American (19 males, 24 females), Asian (5 males, 1 female), or other (22 males, 11 females).

### Macronutrient distribution of the diet at the start and completion of basic combat training

The percentage of total energy obtained from carbohydrate (48.8±8.3%), protein (15.7±3.6%), and fat (35.2±6.7%) was similar between males and females prior to BCT. At the completion of BCT, energy obtained from carbohydrate increased (main effect of time, *P*<0.05) in both males (wk 0: 48.5±8% vs. wk 9: 51.9±6.8%) and females (wk 0: 49.1±8.7% vs. wk 9: 53.9±6.2%). Compared to wk 0, total energy derived from protein remained steady at wk 9, with no differences between men and women. Total energy derived from fat remained stable in males but decreased (sex-by-time interaction, *P*<0.05) in females at wk 9 (33.0±5.3%) as compared to wk 0 (35.4±7%).

### Cardiometabolic risk at the start of basic combat training

Cardiometabolic risk was evident in new recruits at the beginning of BCT (**Table 1**). Nearly one third of recruits reported smoking or engaging in <20 min/d of physical activity prior to BCT, and a third reported a family history of cardiometabolic disease. Fourteen percent of all Soldiers had a BMI in excess of 30 kg/m^2^. However, the prevalence of recruits with a BMI above 30 kg/m^2^ was five times higher (*P*<0.05) in males. More than half of all recruits were not meeting recommendations for total or saturated fat, fiber, or fruit and vegetable intakes, with no differences between men and women. Mean TC (156±29 mg/dL), LDL (93±25 mg/dL), HDL (males: 45±11 mg/dL, females: 54±11 mg/dL), TG (69±35 mg/dL), and glucose (88±8 mg/dL) were within recommended ranges at wk 0. Females had higher HDL, and lower TG (males: 76±39 mg/dL, females: 60±28 mg/dL) and glucose (males: 90±7 mg/dL, females: 85±8 mg/dL) concentrations compared to males (*P*<0.05). However, TC was above recommended levels in 8%, LDL in 39%, TG in 5%, and glucose in 8% of recruits, and HDL was below recommended levels in 33% of recruits. In the cohort of recruits not meeting blood glucose or lipid recommendations, mean TC, LDL, TG, HDL and glucose concentrations were 215±19 mg/dL, 118±17 mg/dL, 186±24 mg/dL, 37±6 mg/dL and 103±4 mg/dL, respectively.

**Table 1:** Percentage of US Army recruits demonstrating health-risk behaviors and having blood glucose or lipid levels indicative of increased cardiometabolic risk before and after basic combat training.

			Demonstrating health risk (%)		
		Week	Male	Female	Total
Risk factor	Health-risk	(0–9)	(n = 84–118)	(n = 67–91)	
Family history^1^	MI, stroke, or diabetes		35	32	34
*Health risk behaviors*					
Smoker	≥1 cigarette/d		24	31	27
Sedentary	<20 min activity/d		32	43	37
*Anthropometric indicators*					
BMI	>30 kg/m^2^	0	22	4^a^	14
*Biomarker* 2 ^*,*^ 3		9	12^b^	0^a^	7^b^
Glucose	≥100 mg/dL	0	11	4	8
		9	5	0^b^	3^b^
TC	≥200 mg/dL	0	8	9	8
		9	3	3	3
LDL	≥100 mg/dL	0	44	31	39
		9	20^b^	21	21^b^
	≥130 mg/dL	0	6	10	8
		9	3	1^b^	2^b^
HDL	M: ≤40 mg/dL,	0	29	38	33
	F: ≤50 mg/dL	9	31	49^a^	39
TG	≥150 mg/dL	0	6	3	5
		9	1	4	2
*Dietary intake* 4					
Total fat	≥35% total kcal	0	50	51	50
		9	42	33^b^	38^b^
Saturated fat	≥10% total kcal	0	63	63	63
		9	51	40^b^	46^b^
Cholesterol	≥300 mg/day	0	43	36	40
		9	70	54^a^^,^ b	63^b^
Sodium^5^	≥2300 mg/d	0	74	49^a^	63
		9	89^b^	72^a^^,^ b	82^b^
Fiber	<28 g/d	0	93	88	91
		9	82^b^	90	85
Fruits and	<5 servings/d	0	79	78	78
Vegetables		9	49^b^	48^b^	48^b^

TC, total cholesterol; LDL, low-density lipoprotein cholesterol; HDL, high-density lipoprotein cholesterol; TG, triglycerides.

1 Myocardial infarction (MI), stroke, cardiovascular operation or diabetes reported in mother or father.

2 National Cholesterol Education Program (NCEP) Expert Panel on Detection, Evaluation, and Treatment of High Blood Cholesterol in Adults (Adult Treatment Panel III) [49].

3 Diagnosis and Management of the Metabolic Syndrome: An American Heart Association/National Heart, Lung, and Blood Institute Scientific Statement [50].

4 Dietary Guidelines for Americans, 2010 [51].

5 Nutrition for Athletic Performance Joint Position Stand [52].

a Fisher's Exact test; different from males, *P*<0.05.

b McNemar's test; different from week 0, *P*<0.05.

### Cardiometabolic risk at the completion of basic combat training

The prevalence of recruits with risk factors associated with increased cardiometabolic risk was decreased at wk 9 as compared to baseline (**Table 1**). Energy obtained from saturated fat decreased (*P*<0.05) while fiber, fruit, and vegetable intake increased during BCT (main effect of time, *P*<0.05). As such, more than half of recruits met recommendations for total fat, saturated fat, or fruit and vegetable intake by the end of BCT. Compared to wk 0, fewer (*P*<0.05) recruits were obese, and fewer (*P*<0.05) had LDL or glucose concentrations that exceeded recommended levels. The prevalence of recruits with elevated TC (8% vs. 3%) and TG (5% vs. 2%) concentrations also declined, though the difference did not reach statistical significance. The prevalence of recruits with HDL levels below recommended levels increased (33% vs. 39%) but did not reach statistical significance.

### Effects of basic combat training on anthropometrics and biomarkers of cardiometabolic risk

Body mass remained stable in women but decreased in men during BCT (sex-by-time interaction, *P*<0.05; **Table 2**). Overall, percent body fat was lower (*P*<0.05) and fat-free mass (FFM) was higher (*P*<0.05) in males compared to females. Percent body fat decreased in both men and women (sex-by-time interaction, *P*<0.05), however, females gained FFM during BCT, whereas males lost FFM from wk 0 to wk 9 (sex-by-time interaction, *P*<0.05).

**Table 2:** Body mass and body composition of US Army recruits during basic combat training.^1^

	Week 0		Week 3	Week 6	Week 9	
*Body mass (kg)* 2						
Male	84.0±16.2		82.3±14.4^a^	81.5±13.0^a^^,^ b	80.3±12.4^a^^,^ b ^,^ c	
Female	66.3±8.3		66.2±7.8	66.8±7.6^b^	66.4±7.4	
*Body fat (%)* 2						
Male	14.3±4.8		-	-	12.3±3.5^a^	
Female	26.6±5.6		-	-	22.8±5.1^a^	
*Fat-free mass (kg)* 2						
Male	71.7±11.4		-	-	70.3±9.4^a^	
Female	48.2±4.8		-	-	51.0±5.3^a^	
*Body fat (%) by BMI* 2	*<30 kg/m* 2	≥*30 kg/m* 2			*<30 kg/m* 2	*≥30 kg/m* 2
Male	12.9±4.0	18.4±4.4^d^	-	-	11.7±2.8	16.9±4.1^a^^,^ d
Female	26.3±5.4	35.9±3.9^d^	-	-	22.9±5.0	n = 0^a^

1 Mean ± SD; n = 100M, 71F.

2 Two-factor mixed model repeated measures ANOVA with Bonferroni corrections. Sex-by-time interaction (*P*<0.05). Different from

a week 0,

b week 3,

c week 6,

d <30 kg//m^2^ *P*<0.05.

**Figure 1:**
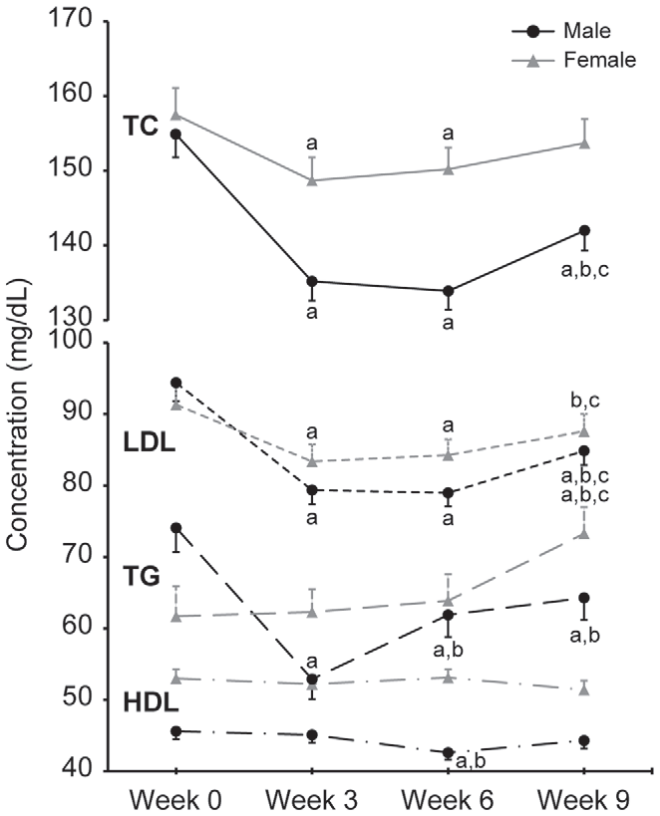
Effect of basic combat training on blood lipids and triglycerides. All values are mean ± SEM; n = 100M, 71F; TC, total cholesterol; LDL, low-density lipoprotein cholesterol; HDL, high-density lipoprotein cholesterol; TG, triglycerides. Two-factor mixed model repeated measures ANOVA. Sex-by-time interaction observed for all biomarkers, *P*<0.05. Different from ^a^week 0, ^b^week 3, ^c^week 6, *P*<0.05.

Sex differences in the responses of TC, LDL, HDL and TG levels to BCT (sex-by-time interaction, *P*<0.05) were observed (**Figure 1**). In men, TC, LDL and TG concentrations decreased (*P*<0.05) by wk 3. Compared to wk 0, TC, LDL and TG concentrations at wk 9 were 8%, 10% and 13% lower (*P*<0.05), respectively. In women, TC and LDL concentrations were below (*P*<0.05) wk 0 values at wk 3 and wk 6, but were not different from wk 0 at wk 9. Triglycerides increased (*P*<0.05) 19% during BCT in women. Although a modest reduction in HDL was observed in men at wk 6, wk 9 HDL concentrations were not different from wk 0. HDL concentrations remained stable in women.

**Figure 2:**
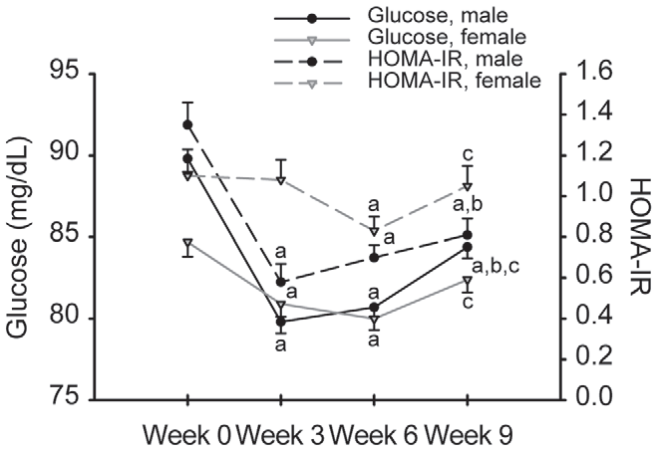
Effect of basic combat training on blood glucose and HOMA-IR. All values are mean ± SEM; n = 100M, 71F. Two-factor mixed model repeated measures ANOVA. Sex-by-time interaction observed for all biomarkers, *P*<0.05. Different from ^a^week 0, ^b^week 3, ^c^week 6, *P*<0.05.

Plasma insulin levels remained stable in women but decreased in men during BCT (sex-by-time interaction, *P*<0.05; **Table 3**). Sex differences in the responses of glucose, and HOMA-IR values (sex-by-time interaction, *P*<0.05) were also observed (**Figure 2**). Glucose concentrations decreased (*P*<0.05) by wk 3 in both men and women. At wk 9, glucose concentrations were 6% lower (*P*<0.05) than at wk 0 in men, but not different from wk 0 in women. In men, HOMA-IR decreased (*P*<0.05) by wk 3 and stabilized before increasing from wk 6 to wk 9. In women, HOMA-IR was below wk 0 values at wk 6, but was not different from wk 0 at wk 9.

**Table 3:** Insulin levels of US Army recruits during basic combat training.^1^

	Week 0	Week 3	Week 6	Week 9
*Insulin (µIU/mL)* 2				
Male^2^	5.9±0.4	2.9±0.4^a^	3.5±0.3^a^	3.8±0.4^a^
Female	5.1±0.5	5.3±0.5^b^	4.2±0.4	5.1±0.4^b^

1 Mean ± SD; n = 100M, 71F.

2 Two-factor mixed model repeated measures ANOVA with Bonferroni corrections. Sex-by-time interaction (*P*<0.05). Different from

a week 0,

b males, *P*<0.05.

## Discussion

As lifestyle behaviors adopted early in life may increase the risk of developing cardiometabolic disease in adulthood, risk factor identification and management in young adults should be a primary focus of long-term disease prevention. To the best of our knowledge, this is the first evaluation of behavioral, demographic, and biochemical indicators of cardiometabolic risk in US Army recruits and the effects of military training on cardiometabolic disease risk factors. Similar to the corresponding civilian demographic, health-risk behaviors and cardiometabolic risk factors were evident in US Army recruits before starting BCT. Most importantly, indicators of cardiometabolic disease risk were diminished after participating in standardized military training, as evidenced by improved lipid profiles, enhanced glycemic regulation, and favorable changes in body composition. These findings emphasize the benefits of standardized military training for reducing common risk factors associated with the development and progression of cardiometabolic disease.

Our laboratory has an established record of documenting the effects of military training on nutritional status indicators of health and performance [38], [39]. However, the current investigation was novel as studies rarely track cardiometabolic risk over time in similar civilian demographics, whereas our study provides a longitudinal evaluation in young adults before, during, and after engaging in a comprehensive training program. We recognize that the collection of data at only one military location, lack of assessments of physical activity, as well as the use of food-frequency questionnaires could be considered limitations that may affect the extension and interpretation of study outcomes. Certainly, characterizing additional subsets of recruits beginning BCT at other training sites would have provided a more representative cross-sectional analysis of cardiometabolic risk and the effects of military training in US Army recruits. Nevertheless, for the first time, the current study determined cardiometabolic risk in new recruits entering military service, which was reduced during structured military training.

The prevalence of recruits reporting health-risk behaviors such as smoking, physical inactivity and poor dietary habits was generally comparable with or slightly greater than findings reported within similar civilian demographics. For example, the prevalence of smoking in recruits (27%) was similar to the prevalence reported in young adults responding to the 2003 Behavioral Risk Factor Surveillance System (29%, BRFSS) [14], and the prevalence of recruits reporting physical inactivity (37%) was similar to the 41% reported in the BRFSS [14]. The percentage of recruits who were obese (14%) was much lower than US population estimates (34%) for adults 20–39 yr [40]. Although obesity rates appear different in recruits, it is important to recognize that new recruits must meet body weight standards in order to enter military service. We also observed LDL concentrations above recommendations and suboptimal HDL in a third of new recruits at the start of BCT. Recent data from Burke et al. [18] and the Tufts University Longitudinal Health Study (TLHS) [19] suggest that the prevalence of lipid abnormalities observed in military recruits was similar to dyslipidemia rates reported in college populations. Moreover, mean TC, LDL and glucose concentrations were not only similar to that reported in the TLHS [19] but lower than those reported in the general US population of adults age 20–29 yr [41]. Taken together these findings indicate that cardiometabolic risk in US Army recruits may be somewhat lower than that of the general civilian population and more comparable to the risk status of college students.

The favorable changes observed in biochemical indicators associated with cardiometabolic risk are likely due in large part to the comprehensive lifestyle modifications required during BCT. These modifications include structured, frequent physical training, and mandatory smoking and alcohol abstention. Physical training has generally been associated with increased HDL concentrations and reduced LDL and TG concentrations [42]–[44]. Specifically, training that elicits an energy expenditure of 1200–2200 kcal/wk has been shown to increase HDL concentrations by 2–8 mg/dL and reduce TG levels by 5–38 mg/dL [45]. Others suggest that the intensity, volume and type (e.g., aerobic, resistance, or combined aerobic and resistance exercise) of physical activity dictates blood lipid responses to training [42]–[44]. In this case, military training incorporates aerobic and resistance training activities across all levels of exercise intensity. Further, the training volume is high, as recent evidence suggests that Soldiers travel approximately 7.5 miles/d during BCT [30]. We suspect that the high levels of energy expenditure associated with the volume of physical activity during BCT and the variety of exercise modalities performed may be the underlying cause for the dynamic decreases in TC, LDL and TG observed within the first 3 wk of training. Although physical activity during BCT may have contributed in large part to the reduction in blood lipids, the overall intensity and volume, which is not prescribed or adjusted based on Soldiers' sex or body mass, may still have been insufficient to elicit an increase in HDL. Nevertheless, the blood lipid and glycemic response is associated with reduced cardiometabolic risk, and interestingly, was more robust in male Soldiers. These findings are at odds with a review from Leon et al. [43], who concluded that the blood lipid responses to physical training occur independent of sex. As such, the sex differences observed in this trial may be related to the higher glucose and TG, and lower HDL observed in male Soldiers at baseline or the absence of weight loss in female Soldiers.

Although the risk of developing cardiometabolic disease in US Army recruits is generally low, the importance of establishing healthy behaviors that affect body weight, body composition, blood lipids, and indicators of glycemic control cannot be understated. Previous studies have established that cardiometabolic disease progression begins early in life [12], [13], [20], [46] and demonstrate the cumulative effects of dyslipidemia through childhood and young adulthood on disease risk in adults [21], [22], [47]. In this study, elevated lipid levels for Soldiers that failed to meet recommendations might be considered biologically insignificant; however, establishing healthy lipid levels early in life may be associated with delayed atherosclerosis and a reduced incidence of cardiometabolic events [48]. As such, our findings, which demonstrate that the lifestyle modifications required during military training favorably influence biomarkers associated with cardiometabolic risk, are clinically significant. Accordingly, military training may mitigate or reverse disease progression if the habits formed during such training are sustained. However, future study is required to determine if Soldiers adopt and maintain the healthy behaviors encouraged during BCT throughout their military careers. Recent reports indicate that Soldiers are making beneficial lifestyle changes after training, yet the number who smoke, are overweight, or maintain suboptimal physical activity and diet regimens remains concerning [27].

In summary, this study demonstrated that the prevalence of health-risk behaviors and biomarkers associated with increased cardiometabolic risk in US Army recruits is similar to that reported in college-age populations at the start of training. More importantly, our findings document the importance of adopting healthy lifestyle modifications for mitigating cardiometabolic risk through improved blood lipids, body composition, and glycemic regulation. Studies to determine if modifications implemented during military training, including structured physical training and improved dietary habits, result in long-term lifestyle adaptations and protection against the development of cardiometabolic disease in active duty military personnel and military veterans are required. Our findings suggest that meaningful changes in cardiometabolic risk profiles in young adults can be achieved in a short period time by consuming a healthy diet and participating in regular physical activity.
